# Could breath analysis by MS could be a solution to rapid, non-invasive testing for COVID-19? 

**DOI:** 10.4155/bio-2020-0125

**Published:** 2020-07-31

**Authors:** Heather J Walker, Michael M Burrell

**Affiliations:** ^1^biOMICS Facility, Department of Animal & Plant Sciences, University of Sheffield, Sheffield S10 2TN, UK

**Keywords:** biomarkers, breath analysis, COVID-19, portable MS

COVID-19 (2019 novel coronavirus) has brought many world-wide challenges in a very short space of time and the race to produce a reliable and rapid test is already on. Currently the test of choice is a PCR method, which is used to detect RNA and therefore genetic information about the virus. However, like all tests there are limitations, this test relies on the person in question actually being infected with the virus and also sufficient virus being present in a swab test [1]. The aim of this commentary is to suggest breath analysis by MS and specifically using mobile MS equipment as an alternative testing method. Using breath as a sample, it would be possible to easily measure volatile organic compounds (VOCs) with MS illustrating an alternative sampling and analysis technique. Breath would not only provide an unlimited sample but also allow noninvasive testing with the possibility of a very quick patient diagnosis by analysis with this method. MS has long been relied upon within a clinical and medical setting, so a move to using it for this application would be a natural step [2].

The reverse transcription polymerase chain reaction (RT-PCR) test already available is reliable and indeed for a test to be most valuable it needs to able to identify the virus before any symptoms are noticeable in a patient. A test that can rapidly check before key workers begin work would give a huge amount of confidence to those in key roles undertaking stressful tasks at this time. As the RT-PCR test is used to detect the presence of an antigen as opposed to antibodies or the body’s immune response, these tests should be able to detect whether the virus is present at an early stage regardless of whether any symptoms are present, and as it is highly specific should allow for few false positive results. If the pattern of who is infected is clear, it becomes much easier to control the spread and quarantine those individuals with links to the virus infection as necessary. This helps Public Health bodies to monitor and limit the spread of the disease better which is critical to lockdown exit strategies around the world and also to rebooting economies.

However, these tests have not been quick enough to meet demand for testing for several reasons. Many hospitals do not have access to PCR machines. In the UK, several distant hubs are available and so samples and/or people need to be transported. PCR currently relies on experienced, trained staff to prepare and analyze samples. The technique potentially has a high rate of false negatives, which can lead to repeat testing or further tests being needed for reliable results [3].

At present the test involves taking nasal and throat swabs which are then sent away for PCR analysis. Clearly there must be sufficient virus for amplification to occur. A false negative can occur for many reasons [3,4]. The sample can contain too little virus because the location swabbed is not appropriate for the stage of infection [5] or the time taken between sampling and analysis is too long. Wikramaratna *et al.* and references therein [4] showed that between day 0 and day 10 after infection; the chance of a positive test declined from 94.39 to 67.15%. Later in disease development the virus multiplies in the lungs but has disappeared from the throat. When this has occurred samples must be obtained from deep in the airways or coughed up sputum. After 4 weeks SARS can be detected in urine [6], however there is little evidence so far that COVID-19 is present in urine [7]. These reasons lead toward the need for an alternative or complimentary technique being available to medical staff and deep breath samples, which are still likely to contain biomarkers for the presence of the virus, maybe the solution.

Currently the testing is done in specialist laboratories using specialist staff, which are often found in large cities or certainly remotely from each other. Testing is not always quickly and easily available remotely. More mobile methods of testing and easy sample collection would hugely increase the capacity to test. Some alternative options are already beginning to be used, such as serology methods or more automated assays, however these are in the early stages of development and home testing kits that are now available still need to be sent to specialist centers for analysis [8]. Targets for testing still need to be increased and made more universally available.

Testing is clearly a necessary objective as countries where the track testing of individuals was introduced early, the benefits have been clearly observed. For instance South Korea who introduced a test and contact tracing promptly have a much lower percentage death rate, around 0.9% per case than the WHO estimation of 3% globally [9]. However, mass testing of populations is not always easy and also depends upon many other environmental factors such as the dynamics of the country, age profile and density of population, which all contribute to the spread and severity of the disease.

## Methodology

The PCR test relies on a swab being taken and particularly on the quality of sample obtained. This should ideally be from the upper respiratory tract with samples of sputum, a mixture of saliva and mucus coughed up from the respiratory tract [10]. Could a simple breath test be a quicker and easier way of sampling as it would be easier to take several replicate samples at the same time?

### Breath analysis

Breath analysis is already well publicized as a suitable sample for the analysis of biomarkers for disease and has been suggested as a suitable sample for detection of COVID-19 [11]. Various breath sampling devices are available such as BioVOC tubes. These are noninvasive methods of sampling and allow for nonmedically trained staff to take the samples [12]. Samples can easily be transferred from the tubes to thermal desorption (TD) tubes, solid-phase microextraction (SPME) or a needle trap device for analysis by GC–MS. TD-GC–MS is already a standardized way of analyzing volatiles in breath and allows for a set amount of gas to be absorbed onto the tube during sampling and desorbed off the tube during analysis. Therefore TD-GC–MS provides a method that can concentrate the sample from a known volume of breath and provide a quantitative result [13]. The recent emergence of portable MS equipment could enhance the capability of testing as it can be easily moved as needed. Sampling using SPME syringes or needle trap devices that could be analyzed by portable GC–MS devices would allow for a rapid analysis, but even sampling using more traditional TD would still allow for easy transfer of samples onto SPME or needle trap syringes. These methods are already well standardized and characterized. Previous studies on various breath sampling methods include that by Lawal *et al.*, who implemented an in-depth study into different breath analysis methods [14], although at present no absolute standard exists.

### Location of testing

One of the key questions is whether breath analysis should be carried out within a laboratory setting with samples shipped and possibly stored before analysis, but this would not give much more of an advantage to this methodology over the current PCR method. With the advent of portable MS, it would make more sense to have the equipment in hospitals, general practioner (GP) surgeries and testing centers and even in airports. It may also be installed in care homes that are particularly vulnerable to the virus and other such locations, and where it would be efficient to do so, kept in a vehicle and transported from one location to another. Mass spectrometers could provide answers in seconds rather than the hours of the current methods. The availability of breath-sample collectors and the ease of use of portable mass spectrometers that can be programmed to look for a biomarker or suite of biomarkers would make it very accessible to nonspecialist users and allow rapid result turnovers, with the possibility of quick further analysis if the result is unclear. This point-of-care approach would be a huge step forward in an increase of testing. The cost–effectiveness of this point-of-care model would need to be taken into consideration.

### Analytical requirements

One of the key aims would be to make sure the volatile components are at a high enough concentration as generally VOCs are only a small percentage of breath as compared with CO_2_. In 2018, Hanna *et al.* conducted a systematic review of breath in relation to the diagnosis of cancer. This suggested that multicenter clinical trials for cancer diagnosis which involved standardized methods of breath collection were required [15]. Another recent study into breath analysis as a means of identifying cancer concluded that the main advantages of breath analysis was that it was noninvasive, it was easy to use, it had prognostic abilities, and it was low cost. However it is acknowledged that it may not yet be a stand-alone diagnostic method and may benefit from being used in combination with currently available methods to increase the accuracy of detection [16].

### SPME as an approach

SPME has several advantages for sampling volatiles from breath; varying levels of moisture do not alter the absorption of volatiles onto the fibres, the volatiles are concentrated in one simple step, the sample can be placed directly into the GC–MS. It is also very cost-effective as the SPME fibres can be used multiple times. The analysis can be done manually using portable equipment or automated using static laboratory equipment. Aksenov *et al.* used various different SPME technologies to illustrate the wide coverage of breath metabolites that could be measured by the combination of SPME and GC–MS [17]. Biomarkers for disease are known to be present in breath [18] and SPME has already been used to detect lung infections [19]. The Torion T9 GC/MS is a portable machine (weighing 15 kg) that will be ready to analyse samples within 15 min of starting up and provides an analysis time of a few minutes. It can be programmed to automatically identify biomarkers of interest allowing a nonspecialist to run the analysis. Therefore, the result can be obtained while the patient waits and where there is doubt about the result a second sample can easily be obtained. As portable mass spectrometers have their own integral gas supply, pump and batteries the equipment needs no specialist services for operation. Thus, rather than shipping samples the equipment itself can be taken to the patients, for example, in care homes.

## Discussion

Exhaled breath contains thousands of VOCs, which may include not only exhaled products but also remnants of the virus itself. To be able to analyze these routinely a metabolomics approach needs to be taken to understand the distribution of metabolites within the samples and to be able to identify significant biomarkers. Breath analysis has routinely been used for detection of alcohol in breath but also in more clinical settings. To assess exposure to the biomarkers medical staff could wear TD tubes. This procedure is already used by veterinary nurses who are exposed to nitrous oxide on a regular basis. Exposure is then measured by GC–MS analysis [20]. Once reliable biomarkers have been established the level of hazard to infection could be monitored in wards. If TD tubes were used for the monitoring the amount of exposure and the pattern over time in relation to the number of patients in the ward could be established. Otherwise an exhaled breath container could be used. Work has already demonstrated that breath exhaled from patients with pneumonia show an altered metabolism for volatile organic compounds [21]. As COVID-19 appears to affect the lungs, particularly if the patient has damaged lungs already through smoking or chronic obstructive pulmonary disease (COPD), these patients are more at risk.

For home collection of samples, it would be much easier for a nonspecialist to take samples by exhaling into a container even if multiple samples were needed, rather than the current sampling technique of taking a single swab of the throat and nose. This is because the best area to swab may change depending upon the stage of infection. A recent study found that nasopharyngeal swabs may be more suitable at later stages of infection than oropharyngeal swabs [22].

The key would be whether there is a particular biomarker that indicates the presence of the virus or whether it is a suite of metabolites and how early a change in metabolism can be identified. To this end work has already begun on a Human Breath Database, which could help define key markers [23]. Using a library-based system with a particular list of biomarkers as targets could provide an easy ‘yes or no’ answer or recommend further testing with swabs collected for analysis by PCR. The ability to automate analysis, the lack of a need for a specialist, the simplicity of the system and its portability would allow rapid expansion in capacity of testing. As indicated previously GC–MS is already used for medical analysis and therefore the equipment would already be available for many other uses.The advantage of MS is that it could highlight more than one problem with the same sample.

## Conclusion

There is much evidence that breath analysis may well be suitable for the analysis of biomarkers. Breath represents a noninvasive, limitless sample that when combined with fast, portable MS methods would make an alternative or complimentary test to the PCR test already available.

Although sample uniformity may need to be addressed the limitless supply of breath is suitable for taking multiple samples and, thus, allowing replication and reducing the likelihood of false positive results like an A and B sample in drug testing procedures currently. Sampling of breath is likely to be much less problematic than swab testing particularly for home sample collection kits due to the fiddly nature of collecting the sample and the importance of avoiding any contamination on the swab from other germs. Evidence is already showing that depending on the stage of infection with the virus that swab tests may not always be the best test [24].

MS is already being used for breath analysis. The key would be to find biomarkers related to COVID-19 and to understand the variability and reproducibility of MS analysis of breath samples infected with COVID-19 and to make sure that any biomarkers were identified with a statistical confidence. Translation of these findings to a routine analysis should be fast.

Studies able to rapidly transpose MS-based breath analysis, using cases at different stages of disease along with controls, from the lab to a clinical setting are what is needed to take this forward.
